# Application of Switchable Hydrophobicity Solvents for Extraction of Emerging Contaminants in Wastewater Samples

**DOI:** 10.3390/molecules25010086

**Published:** 2019-12-25

**Authors:** Guillermo Lasarte-Aragonés, Alejandro Álvarez-Lueje, Ricardo Salazar, Carla Toledo-Neira

**Affiliations:** 1 Departamento de Química Analítica, Instituto de Química Fina y Nanoquímica, Edificio Marie Curie Anexo, Campus de Rabanales, 14071 Córdoba, Spain; b22laarg@uco.es; 2 Departamento de Química Farmacológica y Toxicológica, Facultad de Ciencias Químicas y Farmacéuticas, Universidad de Chile, 8380494 Santiago, Chile; aalvarez@ciq.uchile.cl; 3 Laboratorio de Electroquímica MedioAmbiental, LEQMA, Departamento de Química de los Materiales, Facultad de Química y Biología, Universidad de Santiago de Chile, USACH, 9170022 Santiago, Chile; ricardo.salazar@usach.cl

**Keywords:** emerging contaminants, switchable hydrophobicity solvents, homogeneous liquid-liquid microextraction, high-performance liquid chromatography, partition coefficient

## Abstract

In the present work, the effectiveness of switchable hydrophobicity solvents (SHSs) as extraction solvent (*N*,*N*-Dimethylcyclohexylamine (DMCA), *N*,*N*-Diethylethanamine (TEA), and *N*,*N*-Benzyldimethylamine (DMBA)) for a variety of emerging pollutants was evaluated. Different pharmaceutical products (nonsteroidal anti-inflammatory drugs (NSAIDs), hormones, and triclosan) were selected as target analytes, covering a range of hydrophobicity (LogP) of 3.1 to 5.2. The optimized procedure was used for the determination of the target pharmaceutical analytes in wastewater samples as model analytical problem. Absolute extraction recoveries were in the range of 51% to 103%. The presented method permits the determination of the target analytes at the low ng mL^−1^ level, ranging from 0.8 to 5.9 (except for Triclosan, 106 ng mL^−1^) with good precision (relative standard deviation lower than 6%) using high-pressure liquid chromatography (HPLC) combined with ultraviolet (DAD) and fluorescence (FLR) detection. The microextraction alternative resulted in a fast, simple, and green method for a wide variety of analytes in environmental water sample. The results suggest that this type of solvent turns out to be a great alternative for the determination of different analytes in relatively complex water samples.

## 1. Introduction

Analysis of environmental or biological samples deals very often with complex matrix and the presence of several additional chemical species, which impact significantly on the instrumental determination of the target analytes, in particular, for those at very low concentration level [1,2,3]. Selectivity and sensitivity of most analytical determination can be considerably decreased in such scenarios, especially in routine chromatographic or capillary electrophoresis methods. To overcome those limitations, sample pretreatment steps to isolate and preconcentrate analytes of interest have been implemented in analytical procedures [4].

Ideally, the substeps involved in any sample treatment should be simple, easy to automate, miniaturized, cheap, and safe for the environment and operator [5]. In this context, microextraction (ME) techniques, especially solid-phase microextraction (SPME) [6,7] and liquid-phase microextraction (LPME) [8,9,10], have emerged as alternatives to classical analytical techniques, traditionally associated with tedious steps with great solvent requirements. In the context of this work, LPME has gained interest in the analytical community since the very first approach in the 1990s and liquid continuous development. The analytical community efforts were focused in fast, inexpensive, and reduced solvent-usage alternatives in a miniaturized format [11]. These groups of innovations for more efficient and reduced environmental-impact solvents ultimately led to the dawn of green chemistry and green analytical chemistry [12,13,14]. As a result of this effort came the concept of a solvent in which it is possible to trigger a drastic change in the properties of a solvent; subsequently, the same could be used for several consecutive process steps [15] reducing the overall waste and consumption. Later, the so-called switchable hydrophilicity solvents (SHS) were introduced first by Jessop et al. in the industry context [16,17,18]. This family of solvents, in particular secondary and tertiary amines, could be reversibly switched from being fully immiscible (hydrophobic) to fully miscible (hydrophilic) in aqueous phase. This transition occurs if CO_2_ is solubilized in water and then removed [19]. Jessop et al. established the phase switching behavior as a mechanism of protonation of the amines by carbonic acid, which triggers the change from immiscible to miscible state. In the presence of both CO_2_ and water, the amines are converted into water-soluble bicarbonate salts [17]. The characteristics of this type of solvents have been previously explored in the microextraction context as a homogeneous liquid–liquid microextraction (HLLME) or dispersive liquid–liquid microextraction alternatives [20,21,22,23,24]. HLLME involves the solubilization of an extractant phase on a donor (sample) phase achieving a contact between both virtually infinite [25]. Thanks to the polarity switch, the SHS are excellent candidates for this type of ME procedure [20]. Switchable solvent-based procedures require a minimum, where not inexistent, intervention of external apparatus or complex lab equipment, which combined with the use of CO_2_ as a non-toxic reagent, is a great environmental-friendly alternative. Different applications have been proposed for these solvents in the ME context for different matrices, biological [26], environmental [27], or food and drinks [28]. In general, SHS-based procedures are simple, fast paced (allowing an increased sample throughput), and compatible with a variety of instrumental techniques for a wide range of analytes [29].

The demand of new chemicals for pharmaceutical [30], cosmetic [31], or industrial [32] purposes has a major global impact on water quality in the developed societies. These compounds are considered as contaminants of emerging concern (CECs) and require a strong legislation by agencies [33] to control and prevent its presence in the environment [34]. In particular, NSAIDs, antibiotics, and steroid hormones are of special relevance. For example, NSAIDs, are a group of widely used pharmaceuticals in human and animal care and their presence in the aquatic environment can affect local fauna [35] with non-lethal effect. Anthropogenic antibiotic presence in an aquatic environment has been related to the development of resistance by microorganisms [36]. Steroid hormones can induce feminization, decreased fertility, or hermaphroditism even at low doses [37] in fish. Their origin can be ascribed to human excretion or unused product disposal [38].

Contaminants of emerging concern are present in a variety of waters, at very low concentration levels, making microextraction a great alternative for its sensitive detection. In the present work, an SHS-based ME alternative is presented for the extraction of emerging contaminants from environmental water samples, combined with HPLC-DAD/FLR for selective analyte detection. The presented procedure results in a fast, simple, and green solution for a wide variety of analytes of different polarity with sensitivities in the ng mL^−1^ level with great precision.

## 2. Results and Discussion

### 2.1. Preliminary Study: SHS Selection

**Figure 1:**
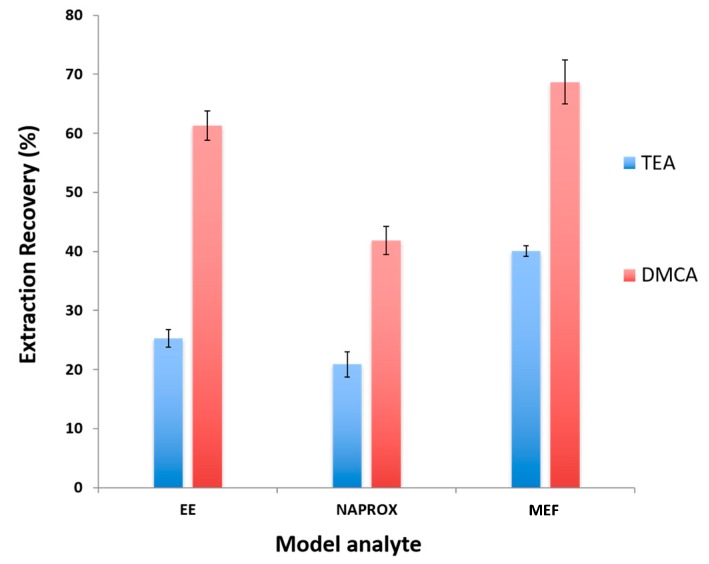
Extraction recovery (%) for the selected target analytes for the two suitable switchable hydrophilicity solvents (SHS) candidates.

Three different solvents were evaluated as potential microextraction SHS candidates: *N*,*N*-Dimethylcyclohexylamine (DMCA), *N*,*N*-Diethylethanamine (TEA), and *N*,*N*-Benzyldimethylamine (DMBA). SHS-HLLME was then performed according to the procedure described in Section 2.4, and the results are presented in Figure 1. DMCA resulted in the highest recoveries for the three model analytes, Naproxen (NAP), Mefenamic acid (MEF), and 17-α-etinil estradiol (EE). By contrast, TEA was unsuitable because of the low recoveries obtained for the three analytes. Furthermore, DMBA resulted in a very poor chromatographic separation when injected, resulting in an unfeasible analyte quantitation. Subsequently, DMCA was selected as an extraction solvent for further optimization of the microextraction procedure.

### 2.2. Optimization of the Microextraction Procedure

A set of parameters impacting extraction efficiency was evaluated in order to optimize the procedure. The selected variables were extractant phase volume; NaOH volume (as phase switching trigger agent); and sample volume. Optimization studies were carried out using 10 mL of aqueous standard containing the target analytes.

#### 2.2.1. Selection of Extractant Phase Volume

**Figure 2:**
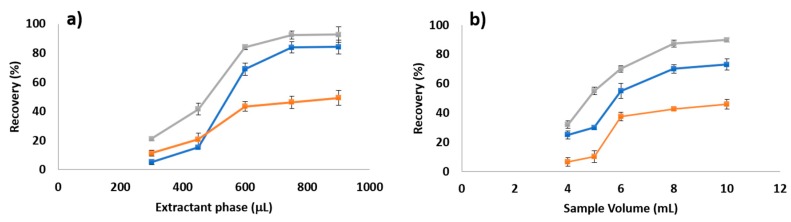

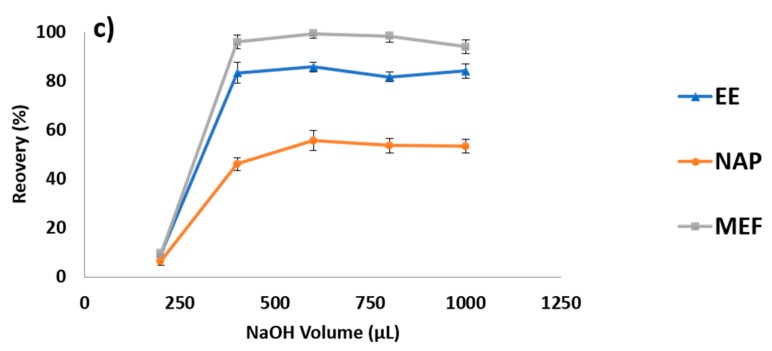
Effect of the extractant phase volume (50:50 SHS:Water) (**a**); sample volume (**b**) and NaOH as phase separation (**c**) trigger on the extraction recovery for a standard solution containing the model analytes at 500 ng L^−1^.

The effect of the volume of extractant phase (300–900 μL) on the extraction performance was evaluated. As observed in Figure 2a, maximum recovery values were obtained for 750 μL of extractant phase (375 μL of extraction solvent), being constant up to 900 μL. Lower extractant phase volumes (300 μL, corresponding to 150 μL of extraction solvent) resulted in an unfeasible phase recovery, and subsequently, poor extraction recovery was observed. As a result, 750 μL of extractant phase was selected for the subsequent optimization experiments.

#### 2.2.2. Selection of Sample Volume

Once the extractant phase was optimized, the sample volume was evaluated. The combined effect of both variables has a critical impact on the preconcentration factors and therefore on the overall extraction efficiency. Sample volume was evaluated between 4 and 10 mL. Figure 2b shows the evolution of the recovery within the range of study. A volume larger than 8 mL has a negligible effect on the extraction recovery, which can be attributed to extractant saturation. As a result, 8 mL was selected as the optimum sample volume. It is worth noting that the larger studied volume was fixed to 10 mL, since it allows the use of standard glass tubes with a neck length that facilitates extractant recovery and use of centrifugation.

#### 2.2.3. NaOH Volume Effect

Phases’ separation is necessary to recover the analytes from the sample in the extractant phase. In the switchable solvent context, different alternatives can be used, but strong pH shifting has been successfully applied in the microextraction context. Different volumes of 20 mol L^−1^ NaOH solution, in the range of 200 to 1000 μL, were studied. Results presented in Figure 2c show a stable recovery above 400 μL for MEF and EE, while 600 μL was necessary for maximum recovery for NAP. According to these results, 600 μL was selected as optimum phase-switching trigger to enhance NAP extraction.

### 2.3. Analytical Figures of Merit

The proposed SHS-HLLME method, once optimized, was evaluated for the determination of eight emerging pollutants (namely, Ketoprofen, Naproxen, Diclofenac, Ibuprofen, Mefenamic Acid, Triclosan, 17-β-estradiol, and 17-α-ethinylestradiol) in water samples. The main figures of merit are summarized in Table 1. A calibration graph was constructed for each analyte by extracting aqueous standards containing the target analytes within the concentration interval shown in Table 1. Different concentration ranges for the analytes were selected according to their different instrumental response (shown in Table 1). Enrichment factors obtained by comparison of calibration graphs before and after extraction were in the range of 8 (for Ketoprofen) to 18 (for Mefenamic Acid). Method sensitivity, expressed as limit of detection (s/n = 3), varied between 0.8 ng mL^−1^ (Naproxen) and 106 ng mL^−1^ (Triclosan). The repeatability of the method was evaluated at ng mL^−1^. Absolute extraction recoveries are in line with other microextraction alternatives [39], ranging from 51% to 103% with great precision (expressed as RSD%) at 20 ng mL^−1^, except for Triclosan, which was evaluated at 500 ng mL^−1^.

### 2.4. Applicability of SHS to Emerging Pollutants Analysis

**Figure 3:**
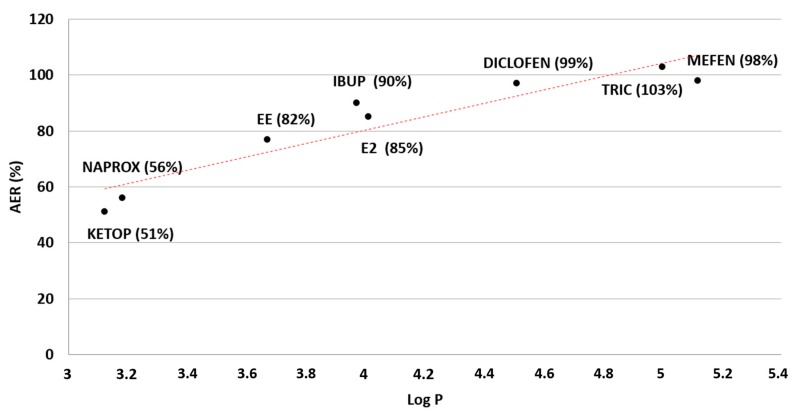
Effect of the target analyte hydrophobicity (LogP) in the absolute extraction recovery (expressed as %) in aqueous samples for *N*,*N*-Dimethylcyclohexylamine (DMCA) as extractant SHS.

The effectiveness of SHS as the extraction solvent for a variety of emerging contaminants was evaluated. Five NSAIDs, two hormones, and an antibiotic were studied as model analytes, with range of Log P values from 3 to 5 (Table 1). According to the results, shown in Figure 3, for analytes with a Log P value greater than 4.0, recoveries from water sample are quantitative, over 85%. The SHS alternates two states, completely hydrophilic and completely hydrophobic. Analytes with lower LogP (more hydrophilic), are expected to be separated from the sample with lower efficiency. For a given analytical problem with known target analytes to be extracted, a graph like Figure 3 can be used as reference. Furthermore, a similar approach can be considered for other switchable solvents in order to evaluate extraction capabilities for a certain analytical problem.

### 2.5. Analysis of Environmental Water Samples

The optimized method was applied to real water samples obtained from the effluent of wastewater treatment plants in Santiago, Chile. Samples were collected as in Section 3.5 and analyzed following the procedure detailed in Section 3.4. No target analytes were detected, and a recovery study was performed at ng mL^−1^ concentration level by triplicate. The results listed in Table 2 show the potential of the proposed SHS-HLLME for the extraction of pharmaceutical compounds from environmental waters. Lowest recoveries can be explained as reflected in Figure 3.

### 2.6. Comparison with Other Microextraction Alternatives

In this paper, an alternative approach to DLLME is presented. In the microextraction context, SHS-HLLME has been introduced as a green alternative that allows infinite contact between phases without any extra solvent or external apparatus [17,20,21]. Regarding the selected analytical problem, pharmaceutical products have become a persistent contaminant, whose distribution profiles in different water compartments are season and location dependent [40,41,42,43]. Evaluation and understanding of this emerging contaminant’s fate and behavior has gained momentum in recent years [44,45]. In this context, a detailed comparison with other published methodological alternatives is presented in Table 3, divided in two great families, steroid hormones [46,47,48,49,50] and NSAIDs [44,51,52,53]. These approaches cover a wide range of extractive phases, including fabric [46], polymers [47], ionic liquids [53], or organic solvents [52]. Our alternative takes advantage of the so-called switchable behavior to avoid the use of organic solvents as disperser agents, minimizing environmental and operator impact. The proposed alternative uses less time per analysis and an overall reduced amount of extractant and sample. In general, it is outperformed, in terms of sensitivity, by those employing mass spectrometry detection [46,49,52], but it avoids the derivatization step [48], in line with the trends of green analytical chemistry [54]. However, when compared with approaches utilizing similar detection methods for the target analytes, it is more sensitive and rapid [44,47] without a complex extraction device assembly [51]. In fact, the phase dispersion and phase´s separation could be carried out without aide of external apparatus [20,21], which may facilitate the application for on-site extraction (i.e., centrifugation speeds up the separation process but can take place during transport of the samples from collection site to analysis facilities). Although its analytical performance is acceptable for the given analytical problem, different approaches can be considered for an extended extraction capability, including larger extractant phase/sample ratio or a mixture of SHSs for a wider LogP/Extraction efficiency coverage [19].

## 3. Materials and Methods

### 3.1. Reagents

All reagents were of analytical grade or better. Sigma–Aldrich (St. Louis, MO, USA) provided the analytes Ibuprofen, Ketoprofen, Diclofenac, Mefenamic acid, Naproxen, 17-β-estradiol, 17-α-etinilestradiol; and SHSs *N*,*N*-dimethylcyclohexylamine (DMCA), Trethylamine (TEA), and *N*,*N*-dimethylbenzylamine (DMBA). Analytes stock solutions (25 µg mL^−1^) were prepared in Methanol and stored at 4 °C. Aqueous standards were prepared by dilution in Milli-Q water as required. Dry ice (Jetcold, Santiago, Chile), as 3-mm-sized pellets, was employed for the solubilization of the DMCA in aqueous phase. A daily prepared 20 mol L^−1^ Sodium Hydroxide (Merck, Darmstadt, Germany) solution was employed to induce the phase’s separation in the extraction procedure. The wastewater (from Santiago, Chile) samples were collected in amber glass bottles and stored at 4 °C until analysis.

### 3.2. Apparatus

Extractant and sample phases were homogenized by means of a Vortex Mixer MX-S (DLab, Riverside, CA, USA). A Hettich EBA 20 centrifuge (Hettich Lab. Technology, Tuttlingen, Germany) was used to reduce the time necessary to complete phase separation during microextraction procedure. The chromatographic analyses were carried out on a Jasco LC Net II system equipped with a quaternary gradient pump (PU-2089 U plus), a Diode Array (MD-2018), a Fluorescence detector (FP-2020), and a column thermostat (CO-2060) (Easton, MD, USA). All analytes were separated by means of a RP Kinetex-Phenomenex (Torrance, CA, USA) C18 column, (150 × 4.6 mm, 5 µm particle size) maintained at 40 °C. The mobile phase consisted of (A) water 0.2% (*v*/*v*) formic acid, (B) methanol, and (C) acetonitrile at a flow rate of 1 mL min^−1^ using a gradient elution profile. The initial composition was fixed at 80:10:10 (A:B:C) and changed to 20:80:0 in 3 minutes. The injection volume was 20 μL. The target analytes were determined using UV-Vis and fluorescence detection. Wavelengths were set at 256 nm for ketoprofen and naproxen; 275 nm for diclofenac and mefenamic acid; and 475 nm for triclosan. Fluorescence detection for ibuprofen was carried out using excitation and emission wavelengths of 220 and 290, respectively. 17-β-estradiol and 17-α-ethinylestradiol were separated using a column temperature of 25 °C and detected by fluorescence with excitation and emission wavelengths of 220 and 290 nm. In this case, the mobile phase composition was (A) water 0.2% (*v*/*v*) formic acid and (B) acetonitrile at a flow rate of 1 mL min^−1^ using an isocratic elution profile of 55:45 (A:B).

### 3.3. Hydrophilic Amine Phase Preparation (SHS)

Equal volumes of SHS and Milli-Q water (100 mL each) were added to 1 L glass bottle with a screw cap; the formation of two phases was observed at this time as a result of the hydrophobic form of the SHS. Then, 10 g of dry ice was added gradually, avoiding the excessive pressure inside the bottle. At this time, a cloudy phase was observed and corresponded to the SHS partially dissolved by the action of carbon dioxide. In order to ensure the solubilization of carbon dioxide, the system was vortex-stirred for 5 min, and this procedure was repeated 10 times, until a single phase formation was observed [20]. The resulting phase, which will be used as extractant phase, consists of a homogeneous 1:1 DMCA:H_2_O mixture.

### 3.4. Microextraction Procedure

A volume of 750 µL of extractant phase was added to a glass test tube with 8 mL of working standard solution or real wastewater sample. The mixture was vortex-stirred by 10 s until a homogeneous phase was observed. Then, 600 µL of a NaOH 20 mol L^−1^ solution was added to the glass test tube, and the apparition of a cloudy phase was observed. After this, the solution was vortex-stirred again by 10 s and subsequently centrifuged at 3000 rpm by 2 min. Finally, 350 µL (out of 375 µL to avoid partial pipetting of aqueous phase) of DMCA were recovered for further analysis. Extraction parameters optimization was carried out by step-by-step variation of extractant phase volume; NaOH volume and sample volume in the ranges referred in Section 2.2. Analysis of aqueous standard containing target analytes at concentration level of 500 ng mL^−1^ was performed in triplicate for each mentioned variable.

### 3.5. Wastewater Collection and Preparation

The proposed method was employed in the analysis of environmental water samples from wastewater treatment plants (WWTPs) from Santiago, Chile. All collected samples were stored in amber glass bottles without headspace and stored at −18 °C until analysis (in triplicate). The WWTPs samples were filtered before analysis using a 2.7 µm Whatman nylon membrane filters, followed by a 1 µm glass fiber filter, and finally 0.45 µm Whatman nylon membrane filter (Whatman, Kent, UK), to prevent the introduction of particulate material in to the HPLC.

## 4. Conclusions

In this article, a switchable hydrophobicity solvent homogeneous liquid–liquid microextraction procedure is presented for the analysis of emerging pollutants and water sample. The proposal uses a reduced volume (375 μL) of *N*,*N*-dimethylcyclohexylamine, a switchable hydrophobicity solvent. As described previously [16], the SHS can be externally controlled to be fully miscible or immiscible with aqueous sample, resulting in a complete separation when it is on its hydrophobic form. Thanks to this, analytes with LogP above a value of 4 can be quantitatively extracted. Analytes with lower LogP values can also be extracted with lower efficiency but enough to be detected with acceptable sensitivity. Furthermore, the range of partition coefficients studied along with their respective extraction efficiency can be used as an indicator in future selection of a certain SHS approach for a given analytical problem. In depth studies can be carried out to generate a matrix comparing values for different solvents and analyte families to select the most suitable extraction solvent.

## Figures and Tables

**Table 1:** Analytical features for the extraction of emerging contaminants by SHS-homogeneous liquid–liquid microextraction (HLLME).

Analyte	Linear Range (ng mL^−1^)	R ^1^	LOD ^2^ (ng mL^−1^)	LOQ ^3^ (ng mL^−1^)	LogP ^a^	AER ^4^ (%)	Precision (Exp. as RSD% ^5^)	EF ^6^
Ketoprofen	100–20,000	0.997	1.9	6.4	3.1	51	3.2	9
Naproxen	100–20,000	0.999	0.8	2.7	3.2	56	4.3	10
Diclofenac	300–20,000	0.998	5.3	17.6	4.5	99	4.1	18
Ibuprofen	150–20,000	0.999	2.3	7.6	4.0	90	5.3	18
Mefenamic Acid	300–20,000	0.999	5.9	19.6	5.2	98	3.4	18
Triclosan	6400–20,000	0.996	106	356	5	103	1.8	18
E2	250–50,000	0.999	3.7	12.2	4	85	3.6	15
EE	250–50,000	0.994	4.5	14.9	3.7	82	6.0	15

^1^ Regresion coefficient; ^2^ Limit of Detection; ^3^ Limit of Quantification; ^4^ Absolute Extraction Recovery; ^5^ Relative Standard Deviation; ^6^ Enrichment Factor; ^a^ data provided by ChemIDplus (SRC Inc.).

**Table 2:** Relative recovery study by SHS-HLLME for the extraction of emerging contaminants in wastewater.

Analyte	Spiked (ng mL^−1^)	Found (ng mL^−1^)	Recovery (% ± SD ^1^)
Ketoprofen	30	17 ± 1	57 ± 5
Naproxen	30	15 ± 1	50 ± 7
Diclofenac	360	350 ± 13	97 ± 4
Ibuprofen	30	27 ± 2	91 ± 7
Mefenamic acid	360	359 ± 15	99 ± 4
EE	30	26 ± 2	85± 7
E2	30	27 ± 1	86± 4
Triclosan	360	360 ± 18	100 ± 5

^1^ SD Standard Deviation.

**Table 3:** Comparison of the proposed extraction procedure with other published methods for the pharmaceutical compounds from water samples.

Extraction Technique	Analytes	Sample Volume (mL)	Extractant Amount	Inst. Technique	LOD ^1^ (ng mL^−1^)	Proc. Time (min)	Notes	Ref.
FSPE ^2^	Steroid Hormones	10	Fabric piece	UHPLC-MS/MS	0.001–0.264	45	Solvent wash fabric reuse	[46]
BAµE ^3^		25	8.1 mg	HPLC-DAD	0.01–0.1	Hours (16.5)	Long procedure	[47]
DLLME		4.5	250 µL (+200 µL disp.)	GC-MS	0.011–0.082	3	Derivatization required	[48]
DLLME		7.5	110 µL (+500 µL disp.)	MEKC ^4^–MS	0.04–1.1	7		[49]
DLLME-SFO ^5^		5	10 µL (+200 µL disp.)	UPLC-UV	0.8–2.7	n/a ^6^		[50]
SHS-HLLME		8	375 µL	HPLC-DAD-FLR	3.7–4.5	2.3		Present method
HF-LPME	NSAIDs	50	20 cm Fiber (+10 µL acceptor phase)	HPLC-MS	7.1–89.3	Hours (n/a)	Long sample preparation	[44]
µLPME		0.005	5.0 µL	HPLC-UV	70–300	5	Microfluidic device	[51]
DLLME		5	200 µL (+1000 µL disp.)	HPLC-DAD-MS	0.65–1.3	15		[52]
DLLME		5	90 µL (+210 µL disp.)	HPLC-UV	17–95	n/d		[53]
SHS-HLLME		8	375 µL	HPLC-DAD-FLR	0.8–5.9	2.3		Present method

^1^ Limit of Detection; ^2^ Fabric Phase Sorptive Extraction; ^3^ Bar Adsorptive microextraction; ^4^ Micellar Electrokinetic Chromatography; ^5^ Single Floating Organic drop; ^6^ not available/disclosed.
